# Triple-Negative Papillary Carcinoma of the Breast: A Case Report

**DOI:** 10.7759/cureus.69020

**Published:** 2024-09-09

**Authors:** Abdulrahman Karmach, Isabelle S Beaudoin, Sarah Navina, Joseph A Di Como

**Affiliations:** 1 Research, St. George's University School of Medicine, St. George's, GRD; 2 Pathology, Banner Health, Phoenix, USA; 3 Breast Surgical Oncology, Ironwood Cancer and Research Centers, Scottsdale, USA

**Keywords:** breast cancer pathology, breast mass, breast oncology, hormone receptor, intraductal papilloma, invasive ductal breast carcinoma, malignant breast masses, papillary carcinoma breast, triple negative breast cancer, triple negative papillary carcinoma

## Abstract

Papillary carcinoma is a rare form of breast malignancy, representing only a small percentage of newly diagnosed breast cancers. Bloody nipple discharge is the most consistent symptom reported among patients. These lesions are visualized histologically as fibrovascular cores lined with proliferating neoplastic epithelial cells. Papillary breast carcinomas are characterized by estrogen receptor (ER), progesterone receptor (PR), and/or human epidermal growth factor receptor 2 (HER2) positivity, allowing for targeted therapeutic approaches with favorable outcomes. Triple-negative papillary carcinoma (TNPC) is a rare variant that lacks this characteristic hormone receptor expression, creating a unique challenge in diagnosis and management. Here, we highlight the case of a 43-year-old asymptomatic female with TNPC following an abnormal screening mammogram that revealed a suspicious mass in the left breast. Surgical excision with clear margins remains the cornerstone of treatment, with adjuvant chemotherapy considered for high-risk cases. As there is limited evidence on the efficacy of targeted therapies and hormone-based treatments, this case analyzes the diagnostic criteria, therapeutic options, and prognosis of TNPC to prompt further investigation into specific treatment strategies.

## Introduction

Despite a 91.2% five-year survival rate, breast malignancy carries the highest incidence rate of newly diagnosed cancers in 2024, as well as being the second leading cause of cancer-related deaths in women worldwide [1]. Papillary carcinoma accounts for only 0.5-2% of breast cancer cases, primarily seen in older women with a median diagnostic age of 63 years [2, 3]. These lesions arise as intraductal papillary growths, marked by the complete absence of a myoepithelial cell layer. According to the literature, 93% of papillary carcinomas exhibit estrogen receptor (ER) positivity, while 73% are progesterone receptor (PR) positive [2]. TNPC stands as a compelling anomaly, as triple-negativity in papillary breast carcinoma is an exceedingly rare occurrence. In contrast, triple negative breast cancer (TNBC) is an aggressive malignancy that accounts for approximately 10-15% of all breast cancers and is more commonly diagnosed in younger women, African American women, and those with BRCA1 mutations [4]. The rarity of TNPC makes it a challenging pathology to readily identify, combining the architectural features of papillary carcinoma with the aggressive behavior and poor prognosis of TNBC.

## Case presentation

**Figure 1:**
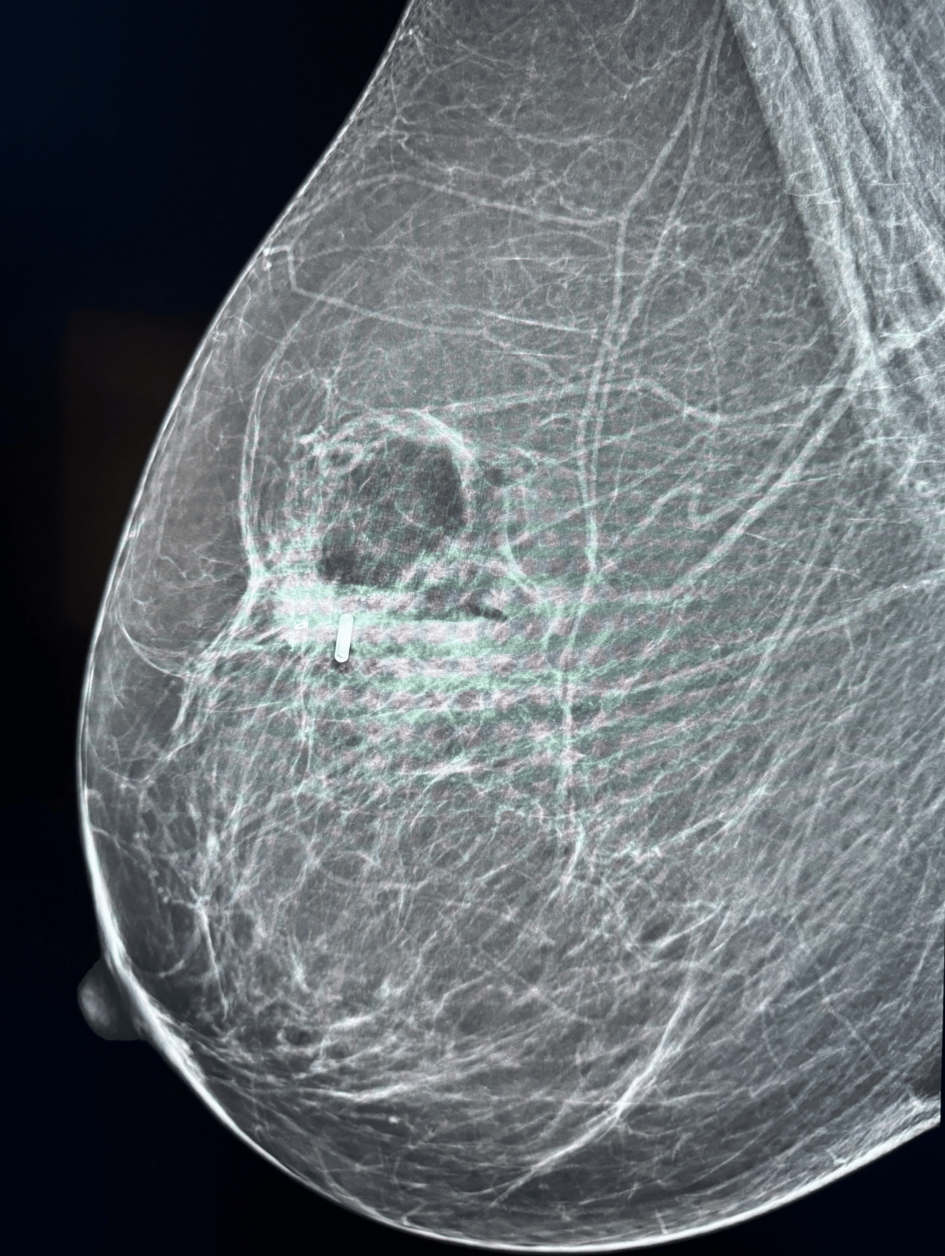
Right mediolateral oblique (MLO) mammography of the left breast demonstrating a 20 mm spiculated mass with irregular calcifications and localization with a radiofrequency tag.

**Figure 2:**
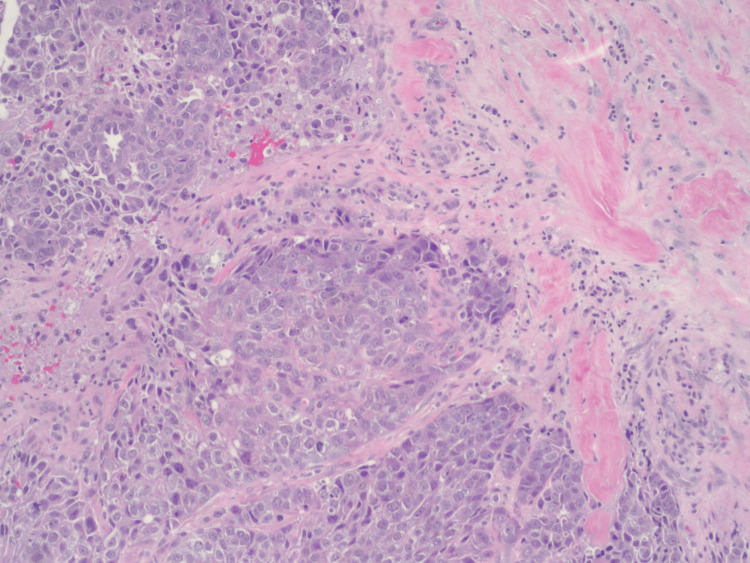
H&E stain of triple-negative papillary carcinoma at 10x magnification.

**Figure 3:**
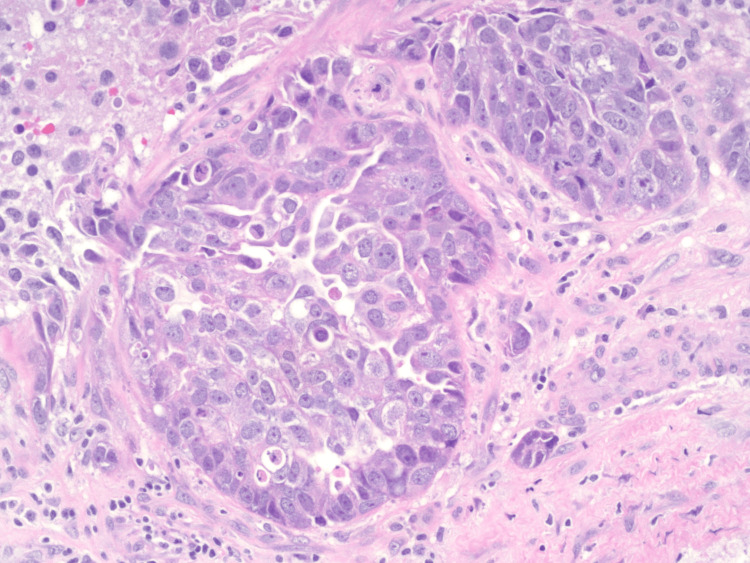
H&E stain of triple-negative papillary carcinoma at 20x magnification.

**Figure 4:**
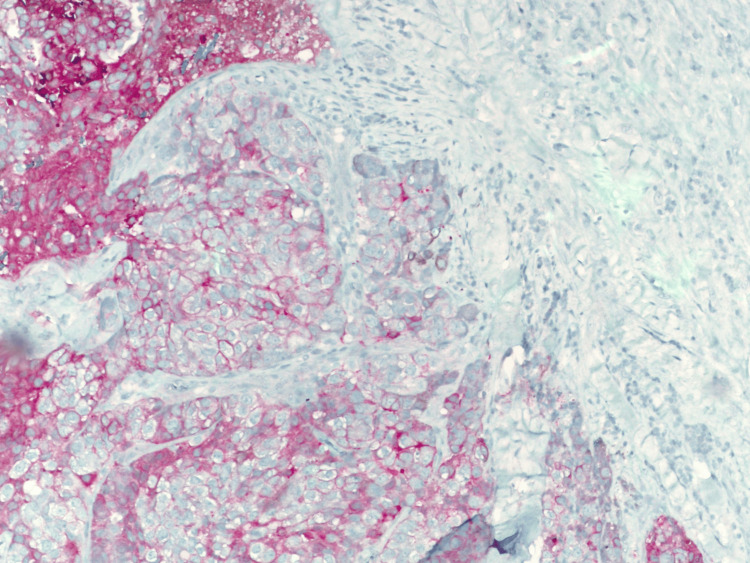
Multiplex IHC stain of triple-negative papillary carcinoma at 10x magnification. IHC: Immunohistochemistry.

**Figure 5:**
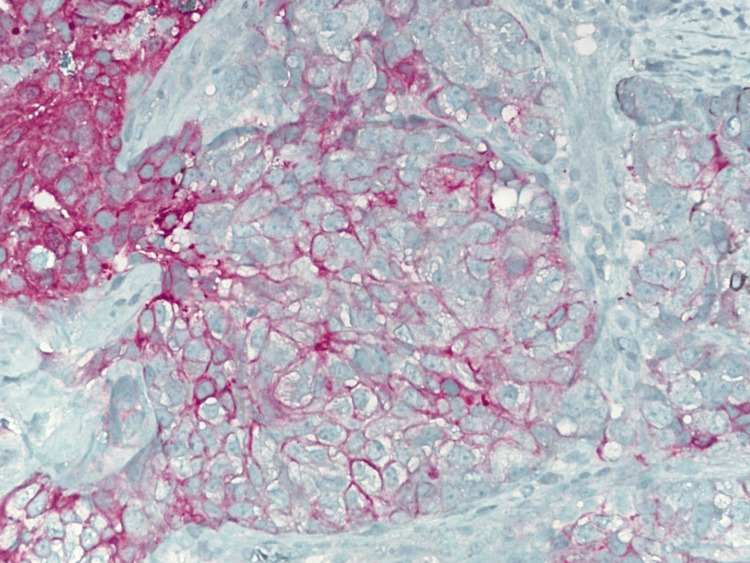
Multiplex IHC stain of triple-negative papillary carcinoma at 20x magnification. IHC: Immunohistochemistry.

A 43-year-old female with no clinical concerns presented with an abnormal screening mammogram demonstrating a mass in the left breast. She subsequently underwent a diagnostic mammogram and ultrasound, which revealed a 20 mm mass in the left breast, for which a biopsy was recommended (Figure 1). Pathology demonstrated an encapsulated papillary carcinoma, intermediate grade, with estrogen receptor 0%, progesterone receptor 0%, and HER2 1+ and negative by fluorescent in situ hybridization. The patient had no significant family history of malignancy, and genetic testing using the Invitae 84-gene oncologic panel was negative for any pathologic variants or variants of unknown significance. She underwent a lumpectomy and sentinel lymph node biopsy. The final pathology demonstrated a 3 mm invasive ductal carcinoma with papillary features and necrosis, grade 3 with high-grade ductal carcinoma in situ measuring 40 x 30 x 20 mm, with clear margins and one sentinel lymph node negative for metastasis. Hormone receptors on the final specimen were ER 0%, PR 0%, and HER2 1+ and negative by fluorescent in situ hybridization. Figure 2 highlights the sheet-like architecture of the lesion with a near absence of tubule formation. The nuclear morphology along with the higher nuclear grade is further appreciated in Figure 3. The absence of basal cells is illustrated on multiplex immunohistochemistry staining, affirming the diagnosis of an invasive carcinoma (Figures 4-5). The specimen underwent a second opinion, which confirmed the findings. The patient underwent adjuvant chemotherapy with four cycles of Taxotere and Cytoxan, followed by adjuvant radiation therapy. She has been followed with high-risk screening protocols including MRI, mammography, annual CT scans of the chest, abdomen, and pelvis, and blood test analysis through the Guardant Reveal Surveillance Program. All subsequent imaging performed one year after surgery demonstrated no areas concerning for metastasis, and Guardant Reveal demonstrated no ctDNA.

## Discussion

This case sheds light on a rare event of papillary breast carcinoma with triple-negativity. Papillary breast lesions characteristically present with unilateral bloody nipple discharge or an abnormal palpable mass, with or without associated pain. Bloody nipple discharge is the most common symptom reported, as seen in over a third of reported cases [5]. For the initial diagnostic evaluation of triple-negative papillary carcinoma (TNPC) of the breast, standard imaging techniques are commonly employed. Mammography often reveals TNPC as an irregularly shaped, high-density mass with indistinct or spiculated margins [6, 7]. Ultrasound is frequently used to provide additional detail, often showing a hypoechoic mass with irregular borders and posterior acoustic enhancement, and detection of cystic components. Breast MRI typically shows a mass with rim enhancement, high T2 signal intensity, and rapid initial enhancement followed by washout, characteristic of TNBC [8, 9]. In the case of TNPC, breast imaging lacks specificity and must be validated with histopathological and immunohistochemical analyses to establish the diagnosis [9].

Papillary carcinoma is characterized by distinct papillary structures within the tumor surrounded by an epithelial cell-lined fibrovascular core. This epithelial lining commonly consists of uniform, layered columnar cells; however, variations such as solid, cribriform, or micropapillary growth patterns may also be present [10]. Invasive features are marked by the absence of the myoepithelial cell layer and extension beyond the fibrous capsule of the lesion, often eliciting a stromal reaction [1, 10]. TNPCs exhibit such patterns in combination with those seen in TNBCs, which demonstrate solid, trabecular, or cribriform cell growth with abundant mitoses, areas of necrosis, and lymphocytic infiltration [10]. With the total absence of hormone receptor expression, immunohistochemistry (IHC) plays a crucial role in the diagnosis of TNPC. As an invasive papillary carcinoma, it lacks positivity for myoepithelial cell markers, such as p63, calponin, and smooth muscle myosin heavy chain (SMM-HC). TNPC often demonstrates positivity for the IHC markers seen in TNBCs, which include CK5/6, CK14, and estimated glomerular filtration rate (eGFR). Expression of Ki-67, an indicator of cancer cell proliferation, is positive in a majority of these lesions and is widely accepted as a prognostic factor used to guide treatment [11].

Papillary carcinomas of the breast generally exhibit indolent behavior without overt invasive or metastatic features [5]. Preferred management typically involves surgical excision followed by radiation therapy and/or adjuvant hormonal therapy. The excision approach may vary from lumpectomy to total mastectomy depending on the specific lesion size, location, and lymph node involvement [12]. Performing surgery first can help determine the appropriate adjuvant treatment by confirming the pathology, including the invasive component, hormone receptor status, and lymph node status, which are critical for tailoring further therapy. The use of adjuvant chemotherapy is generally discouraged in the treatment of papillary lesions, especially those with favorable features such as hormone-receptor positivity [12]. Conversely, chemotherapy serves as the initial treatment method for TNBC, followed by surgical resection. Neoadjuvant chemotherapy is employed to reduce the size of the tumor, allowing for breast conservation with partial mastectomy or lumpectomy [13]. In this patient presenting with TNPC, a multifaceted approach to treatment was required, and adjuvant chemotherapy was implemented following surgery. Pathological complete response (pCR) following neoadjuvant chemotherapy has been associated with improved long-term outcomes and offers insights into disease-free survival rates [13]. Standard regimens include anthracyclines and taxanes, which have shown efficacy in reducing recurrence rates [14]. Platinum-based chemotherapies have also been effective in patients with BRCA1/2 mutations [13, 14]. The significant treatment challenge of TNPC is that the coexistence of papillary carcinoma and a triple-negative phenotype in the breast is exceedingly rare, which limits available management protocols. Surgical management remains the gold standard, and given the triple-negative status, adjuvant therapy would likely necessitate the use of systemic chemotherapy tailored to TNBC protocols. Strategies that integrate approaches for both papillary carcinoma and TNBC may be effective for TNPC and call for further investigation.

## Conclusions

TNPC present a unique challenge to clinicians, blending the architectural characteristics of papillary carcinoma with the aggressive molecular profile of TNBC. This rare convergence necessitates a multidisciplinary approach that integrates advanced imaging techniques, precise histopathological analysis, and tailored therapeutic strategies. Surgical excision remains the primary management strategy for TNPC, often followed by adjuvant chemotherapeutics employed in TNBCs to mitigate the high risk of recurrence. Further research is essential to enhance our understanding of TNPC's pathophysiology and optimize therapeutic interventions, ultimately improving outcomes for individuals facing this challenging diagnosis.
